# Formation, Contraction, and Mechanotransduction of Myofribrils in Cardiac Development: Clues from Genetics

**DOI:** 10.1155/2012/504906

**Published:** 2012-06-10

**Authors:** Javier T. Granados-Riveron, J. David Brook

**Affiliations:** Institute of Genetics, Queen's Medical Centre, School of Biology, University of Nottingham, Nottingham NG7 2UH, UK

## Abstract

Congenital heart disease (CHD) is the most common birth defect in humans. It is a leading infant mortality factor worldwide, caused by defective cardiac development. Mutations in transcription factors, signalling and structural molecules have been shown to contribute to the genetic component of CHD. Recently, mutations in genes encoding myofibrillar proteins expressed in the embryonic heart have also emerged as an important genetic causative factor of the disease, which implies that the contraction of the early heart primordium contributes to its morphogenesis. This notion is supported by increasing evidence suggesting that not only contraction but also formation, mechanosensing, and mechanotransduction of the cardiac myofibrillar proteins influence heart development. In this paper, we summarize the genetic clues supporting this idea.

## 1. Introduction

The heart is the first organ to form in vertebrates during embryogenesis, and cardiac contraction initiates shortly after the cardiac tube is formed. The ever increasing needs of the peripheral tissues of the growing embryo for nutrients and oxygen are met gradually by the blood circulation induced by early cardiac contraction. The heart primordium has to perform its function for most of its own development, and this unique feature has profound implications in the relationship between form and function during cardiogenesis: factors that compromise early cardiac function result in altered morphology, and heart malformation usually has an important impact over the function of the organ. Defective heart development results in congenital heart disease (CHD), the commonest congenital defect in humans and a leading cause of infant mortality worldwide. CHD is a complex disease, as multiple genetic and environmental factors have been implicated in its pathogenesis. As in most complex diseases, most cases are sporadic, although a relatively small fraction of cases show familial segregation, following Mendelian patterns of inheritance. The study and identification of the genes causing the familial forms of the disorder can also give important information about the genetic component of the more common, complex variety.

Genetic linkage is a technique that identifies genes responsible for inherited traits solely based in their position on chromosomes without prior knowledge of their product or its function. More than two decades ago, by genetic linkage analysis, a mutation of a gene encoding a myofibrillar protein (*β*-cardiac myosin heavy chain, MYH7) was shown to cause another cardiac disease, hypertrophic cardiomyopathy (HCM) [1]. Since then, mutations in multiple genes encoding myofibrillar proteins have been identified as responsible for other cases of this disease and other types of cardiomyopathy: dilated cardiomyopathy (DCM), arrhythmogenic right ventricular cardiomyopathy (ARVC), and restrictive cardiomyopathy (RCM) (for a recent review, see Ghosh and Haddad, 2011) [2].

The first genes shown to be responsible for familial cases of CHD (*NKX2-5* and *GATA4*) encode transcription factors. Subsequently, mutations in genes that encode other types of protein, like signalling and structural molecules, have been described in families with Mendelian CHD, and common variants have been identified as predisposing factors for non-Mendelian CHD in large cohorts of sporadic cases (for a review, see Wessels and Willems, 2010) [3].

Some relatively recent developments in the search for the genetic origins of CHD were initiated by an unexpected observation: a causative point mutation in chromosome 14 was identified in a large family with an autosomal dominant atrial septal defect (ASD), one of the most common forms of CHD [4]. This mutation is located in a gene that encodes a myofibrillar protein (*α*-cardiac myosin heavy chain, MYH6). Subsequently, we and others have shown that mutations in other genes that encode other myofibrillar proteins can cause defective cardiac development [5–10]. Traditionally, compromised contractility of the cardiac muscle by the presence of dominant-negative mutant myofibrillar proteins or their haploinsufficiency (for nonsense mutations) was expected to cause only functional phenotypes during postnatal life which in turn induce a maladaptive response, as occurs in the different types of cardiomyopathy. The unexpected observation that mutations in these genes can also affect the functionality of the heart during its early development and thus induce cardiac malformation is supported by analysis of spontaneous and induced mutations in model organisms.

In this paper, we discuss recent findings that indicate the importance of the functionality of the myofibrils, specifically their formation, contractility and mechanotransduction, in several aspects of cardiogenesis.

## 2. Overview of Cardiac Development

Cardiac development is a complex process, by which an initial primordium consists of a straight tube of cardiac muscle (myocardium), lined by a single layer of endothelial cells (endocardium) [11], bends to the right (looping) in order to define the prospective chamber that receives blood through the veins (primitive atrium) from the chamber that expel blood to the arteries (primitive ventricle) [12]. The extracellular matrix that separates myocardium and endocardium (cardiac jelly) expands to form the endocardial cushions, located between the prospective atrium and ventricle and between this and the prospective outflow vessel [13]. The myocardium and endocardium forming the wall of the future chambers expand (ballooning) [14] and penetrate the interior of the chambers with ramified ridge-like structures called trabeculae [15]. Both the primitive atrium and ventricle are divided into right and left chambers by atrial and ventricular septation, respectively, resulting in the adult configuration of a right and left atria and a right and left ventricle, and each ventricle is aligned with its atrial counterpart [16]. Both the endocardial cushions and the trabeculae contribute to both atrial and ventricular septation. Additionally, the endocardial cushions condense to form the valves between atria and ventricles and between the ventricles and the prospective outflow vessels. Trabeculae also form the papillary muscles that anchor the valves to the inner surface of the ventricles [17].

## 3. Defective Myofibrillogenesis in the Heart Causes Cardiac Malformation

**Figure 1:**
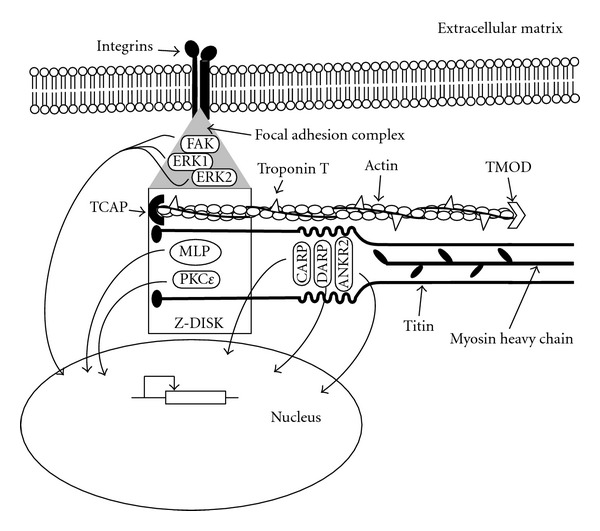
Schematic representation of the structures of the myofibrils responsible of contraction, sensation, and transduction of mechanical stimuli in the developing heart. Force is generated by the thick filaments formed by myosin heavy chain (amhc, MYH6, MYH7) associated with myosin regulatory (Myl7) and essential light chains and their interaction with the thin filaments, formed by actin (ACTC1, cfk) and troponin T (Tnnt2), TCAP and TMOD, amongst others. Titin spans a half of the sarcomere, from the Z-Disc (where it interacts with TCAP and MLP) to the M-band. One of its intermediate segments, the N2A-region, acts as a mechanotransducer and binds CARP, DARP, and ANKR2. The peripheral myofibrils of the cardiomyocytes are linked to integrins embedded in the cell membrane (sarcolemma) by means of the focal adhesion complexes of the costameres, formed by FAK ERK1 and ERK2, amongst others. In response to mechanical stimulation, proteins bound to titin (CARP, DARP, and ANKR2), proteins of the Z-Disc (MLP, PKC*ε*), as well as FAK, ERK1, and ERK2, located to the focal adhesion complexes, translocate to the nucleus, where they interact with cardiac transcription factors to modify gene expression.

*In vivo* studies in chicken and mouse have shown that tropomodulin is an essential component of the myofibrils required for its proper formation [18, 19]. This protein is located in the membrane of the differentiating myocardial cell, closely associated with spectrin [18]. Once tropomyosin localizes within the thin filament, tropomodulin localizes in the pointed end of the actin filament, where it is thought to act as length stabilizer (Figure 1) [20].

Mice with targeted mutations of Tmod1 exhibit disruption of myofibrillogenesis, absence of cardiac contraction, looping defects, trabeculation defects, thinning of the ventricular wall, and impaired chamber formation [19, 21, 22].

## 4. Mutant Thick and Thin Filament Proteins and Cardiogenesis: Lessons from Animal Models

Analysis of spontaneous and experimental mutations in animal models has highlighted the role of myofibrillar proteins in cardiogenesis. In vertebrates, contraction of the cardiac primordium through interaction between the thick and thin filaments of the sarcomere occurs very early in development, well before the circulation of blood is needed to sustain the tissues of the embryo. It has been proposed that this initial activity of the cardiac myofibril apparatus functions as a stimulus for adequate heart development [23]. Deficiency of the genes encoding proteins of both the thick and thin filaments of the sarcomere has been implicated in cardiac malformation. For example, in the case of proteins of the thin filament, mutations in the genes encoding cardiac troponin t (*tnnt2*) and sarcomeric actin (*cfk*) in zebrafish induce endocardial cushion and valve formation defects [24]. Targeted disruption of the cardiac troponin T gene (*Tnnt2*) causes looping and endocardial cushion defects in mice [25]. In the case of genes encoding proteins of the thick filament, mutation of the atrial myosin heavy chain gene (*amhc*) results in deficient ventricular development [26]. Defective atrial myosin heavy chain causes inadequate atrial septation in chicken (MYH7) [27] and malformation of the valves and trabeculae in the amphibian *X. tropicalis* (myh6) [28]. Abnormal cardiac chamber morphology, possibly due to inadequate looping of enlarged and amorphous heart tubes, was detected in mice with mutant atrial myosin regulatory light chain (*Myl7*) gene [29].

## 5. Mutations of Myofibrillar Protein Genes in CHD

The aforementioned finding by linkage that a mutation in the *α*-myosin heavy chain gene (*MYH6*) causes a dominant form of ASD [4] has been supported by reports of additional mutations in the same gene in cases of ASD and other types of CHD by us [6, 7] an others [10]. Recently, by array-based sequence of 13 sarcomeric genes in 31 familial cases of ASD, *MYH6* was identified as the predominant sarcomeric disease gene for ASD [10]. Additionally, mutations of the *α*-cardiac actin gene (*ACTC1*) have been identified as causative in other familial and sporadic cases of ASD [8]. Different mutations of the *ACTC1* gene have been found in cases of septal defects associated with ventricular noncompaction [9] (expansion of the trabeculated component of the ventricular wall at the expense of its compact layer). In cases of another type of CHD (Ebstein's anomaly) associated with ventricular noncompaction, mutations of the *β*-myosin heavy chain gene (*MYH7*) have been identified [5]. Other mutations of *MYH6*, *ACTC1*, and *MYH7* also cause hypertrophic and dilated cardiomyopathy [2, 30–32].

## 6. Mechanotransduction of Myofibrils and Heart Development

Besides their function in contraction, specific myofibrillar and associated proteins can also act as sensors and transducers of the strains imposed by their own activity and the resulting fluid dynamics. The function of some of these mechanosensors and transducers could be relevant for cardiac development.

Titin, the largest myofibrillar protein, spans half of the sarcomere as it links its outer limit (the Z-band) with its centre (the M-band). This protein is composed of a series of springs of variable resistance that provide elasticity to the myofibrils and also contains segments that act as mechanosensors [33, 34]. The Ig-domains 180 and 181 in the N2A-region of titin interact with three proteins of the muscle ankyrin-repeat family, CARP (also known as ANKRD1 and a known target of NKX2.5 [35] and other cardiac transcription factors [36]), DARP, and Ankrd2 [37, 38]. When stretch is applied to cardiomyocytes, CARP translocates to the nucleus, where it interacts with transcription factors to modify gene expression [39]. A mutation on the *ANKRD1* gene that enhances the stability and potentiates the transcriptional repression of the cardiac specific *ANF* promoter was described in a patient with total anomalous pulmonary venous return (TAPVR, another type of CHD, where the pulmonary vein empties in a vessel or cardiac chamber different from the right atria). A chromosomal translocation with a breakpoint near the *ANKRD1* gene was discovered in another patient with the same abnormality [40].

Mechanical stimuli are first transmitted to the membrane of the cardiomyocytes (sarcolemma) through the extracellular matrix. Myofibrils are bound to the membrane of cardiomyocytes via specialized focal adhesion complexes concentrated in the costameres. These structures link the sarcolemma to the Z-bands of the sarcomeres that form the peripheral myofibrils [41]. The mechanotransduction of the focal adhesion complexes at the costameres includes phosphorylation of focal adhesion kinase (FAK), extracellular signal-regulated kinases 1 and 2 (ERK1 and ERK2), and paxillin (PXN) (Figure 1) [42].

It has been shown that the focal adhesion complexes at the costameres can react differentially according to the rate and direction of the strain applied to the cell. Prolonged static strain over cardiomyocytes causes FAK to translocate from the focal adhesion complex to the nucleus [42]. Transverse strain (perpendicular to the sarcomere) increases the phosphorylation of FAK, ERK1, and ERK2 in comparison with nonstrained cells, whereas longitudinal strain (parallel to the sarcomere) induces the phosphorylation of ERK1 and ERK2 but does not increase phosphorylation of FAK [42]. It has been proposed that transverse strain extends the FAK molecule, promoting its activation by phosphorylation [43].

Deficiency of FAK in produces a general defect of mesoderm development in mice [44] and, specifically in the developing heart, results in compromised migration of neural crest cells to the cardiac outflow tract, which in turn induces malformation of conotruncus [45]. However, myocardium-specific ablation of the *FAK* gene induces ventricular septal defects (VSDs) and deficient cardiomyocyte proliferation in mice, which suggests that the role of FAK as a mechanotransducer is also important for cardiogenesis and specifically for ventricular septation [46]. Knockdown of *FAK* transcripts by antisense morpholino induced looping defects in *X. laevis *[47]. Upon activation by phosphorylation, ERK1 and 2 translocate to the nucleus [48] and are known to participate in several processes important for heart development, including FGF, BMP, and VEGF signalling [49]. Moreover, a 1Mb microdeletion in chromosome 22q11.2 spanning the *ERK2* gene has been described in patients with VSD and PTA [50]. Ablation of the *PXN* gene in mice results in abnormal heart structure [51]. These data suggest a possible mechanism by which cardiac contraction and the resulting circulation of blood in early development can, in turn, promote heart development by inducing cardiomyocyte proliferation.

In contrast, animal models of mutations of genes encoding other proteins known to act as mechanosensors in the cardiac Z-band and the costameres do not display cardiac malformation. In response to variations in mechanical stress, muscle LIM protein (MLP) translocates to the nucleus [52], where it has been proposed to bind cardiac transcription factors like GATA4 [53]. Also, it has been hypothesised that MLP forms a complex with telethonin (TCAP) and the N-terminal Z1 and Z2 domains of titin that acts as a stretch sensor in cardiomyocytes [54]. Protein kinase C epsilon (PKC*ε*) is necessary and sufficient to induce FAK autophosphorylation [55], and it is known to localize to the Z-band in cardiomyocytes [56] and to translocate to the nucleus in response to pressure overload [57], where it has been suggested to regulate the transcription of *α*-skeletal actin [58]. Humans or mice harbouring mutations of the genes encoding MLP [54, 59], TCAP [60, 61], or PKC*ε* [62] do not show cardiac malformation. Further research is required to establish why defective specific mechanosensing pathways of the myofibrils and associated structures have a deleterious effect in cardiogenesis and others do not.

## 7. Conclusions

Mutations in many genes that encode proteins involved in the formation, contraction as well as force sensing and transduction of the myofibrils produce phenotypes that include impaired cardiac development in animal models and humans. It is being increasingly recognized that the early activity of the embryonic heart has a significant impact in the morphogenesis of the organ. The sensing and transduction of the forces it generates modify the gene expression of the early myocardium, which elicits remodelling, proliferation, and possibly apoptosis. Thus, we suggest that gene candidate approaches designed to discover new genetic determinants of Mendelian CHD or common variants predisposing to the nonfamilial CHD should consider genes involved in these signalling pathways.
